# Kinematic Analysis of the Instep Kick in Youth Soccer Players

**DOI:** 10.2478/hukin-2014-0063

**Published:** 2014-10-10

**Authors:** Alen Kapidžić, Tarik Huremović, Alija Biberovic

**Affiliations:** 1 Faculty of Physical Education and Sports, University in Tuzla, Bosnia and Herzegovina.

**Keywords:** knee angle, biomechanics, velocity, foot, support leg, kicking technique

## Abstract

We attempted to establish which applied kinematic variables significantly contributed to the efficiency of the instep kick motion in soccer. The study sample comprised 13 boys (age: 13 ± 0.5 yrs; body mass: 41.50 ± 8.40 kg; body height: 151.46 ± 5.93 cm) from the FC Sloboda school of soccer. Each participant performed three kicks with maximum strength that were video recorded with two synchronized cameras (Casio Ex-F1) positioned 12 m away from the place of the kick. Data were collected by analyzing the video recordings of each kick. Data processing was performed using the APAS motion analysis system (Ariel Dynamics Inc., San Diego, CA). On the basis of the forward selection method of multiple regression analysis, we determined the correlations between the prediction variables and the selected criteria (speed of the ball; p = 0.01). On the basis of the regression coefficients, it was concluded that two variables significantly contributed to the speed of the ball: speed of the foot of the kicking leg at the time of contact with the ball (p = 0.01) and the distance between the angle support leg and center of the ball (“foot posterior displacement”) (p = 0.01). In order to achieve the best possible technical performance and, therefore, a higher speed of the ball, soccer players must pay attention to two important elements during training. First, it is necessary to position the support leg as close to the ball as possible and, second, maximize the force used in the initial phases of the kick to achieve a high speed of the kicking foot.

## Introduction

Variations of the instep kick are often used in soccer, such as when passing the ball at medium and long distances, when shooting at the goal, and when performing penalty kicks (Kellis and Katis, 2007). Coaching experience and knowledge of a mechanical model of desired performance are necessary for a coach to correct performance among players (Smith et al., 2006). The biomechanics of kicking in soccer is particularly important for guiding and monitoring the training process. Studies in the biomechanics of instep kicking have focused on numerous variables in different populations, but all seek to establish optimal variables, or variables that are most predictive of success, which is most typically defined by the resulting ball velocity (Ismail et al., 2010).

The instep soccer place kick is one of the most analyzed kicking actions in soccer (Dorge et al., 2002). Considering its complexity, application in the game, multiple advantages, and the desire for the best possible technical performance, the instep kick is the subject of much research that has involved all levels of players, from youth athletes to experienced professionals (Ismail et al., 2010; Barfield et al., 2002; Shan and Westwrhoff, 2005; Reilly, 2003; Kellis et al., 2004). Biomechanical techniques are important tools for many sport disciplines, but, in soccer, they are particularly useful for defining the characteristics of skills, improving mechanical effectiveness in execution, and identifying factors that influence successful performance. Knowledge and understanding of biomechanics can enhance learning and performance of sport-specific skills (Ismail et al., 2010; Amiri-Khorasani et al., 2010). Specifically, systems for the kinematic analysis of human movement provide precise measurement of values and parameters of athletes’ movements during performance of any sport technique. Understanding of the biomechanics of kicking in soccer is important for monitoring and correcting performance during the training process (Meamarbashi and Hossaini, 2010). The speed of the ball in an instep kick depends on several factors, including speed of the foot of the kicking leg before contact with the ball, body posture at the moment of kicking the ball, length of the run up to the ball and its angle (Barfield et al., 2002; Stankovic et al., 2004; Meamarbashi and Hossaini, 2010; Dorge et al., 2002).

When using their preferred leg to perform an instep kick, soccer players practise a straighter approach to the ball, place their standing foot closer to the ball, and kick the ball with greater pelvic tilt and greater knee extension of the kicking leg. Variations in the placement of the support leg impact the speed of the ball (Harrison and Mannering, 2006; Kellis et al., 2004; Orloff et al., 2008). These mechanical characteristics of the foot and the ball impact the coefficient of elasticity, which also influences the speed of the ball (Andersen et al., 2008). During a soccer game, at the moment the ball is kicked at the rival’s goal, the efficiency of the kick depends on the conformation of body posture relative to the path of the oncoming ball. For a hard kick, such as a penalty kick or goal kick, two basic mechanical elements must be considered: swinging of the leg to accelerate the foot and the brief interaction of the foot with the ball. The motion of the foot takes roughly one-tenth of a second and the impact lasts for one one-hundredth of a second. For the fastest kicks, the foot must be given maximum speed in order to transfer a high momentum to the ball (Wesson, 2002). The speed of the ball also depends on muscle strength, and the muscle potential is conditioned by the angle of running to the ball (Masuda et al., 2005). The muscles accelerate the thigh, pivoting it about the hip, and accelerate the calf and the foot. As the foot approaches impact with the ball, the leg straightens and, at impact, the foot is locked firmly with the leg. If the interaction of the foot with the ball was perfectly elastic with no frictional energy losses, the speed transferred to the ball would simply follow two laws of conservation: conservation of energy and conservation of angular momentum. As noted, the process of training proper soccer technique is a difficult task (Wesson, 2002).

Due to the complexity of the instep kick, youth soccer players must pay attention to several technical elements during the training process. Biomechanical analyses of the movement are necessary to improve performance of the instep kick, to understand the results of the kick, and to develop new and more efficient movement techniques.

Very few studies of the instep kick have been conducted on youth soccer players in Bosnia and Herzegovina, so we chose this movement for our analysis. The aim of this study was to establish which kinematic variables significantly increase the speed of the ball during the instep kick. Therefore, kinematic variables were evaluated to estimate the position and speed of the kicking leg, the position of the support leg, and body posture during the instep kick. The information gained through this research will contribute to developing a more efficient movement for the instep kick in soccer. Finally, our data will encourage a faster and more efficient training process for the instep kick for youth soccer players, which will lead to an efficient application of the technique in more complex situations.

## Material and Methods

### Participants

The study sample comprised 13 youth soccer players. The average age of the participants was 13 ± 0.5 yrs, body height was 151.46 ± 5.93 cm, and body mass was 41.50 ± 8.40 kg. All of the subjects were students of the FC Sloboda school of soccer. Each participant performed three full-force kicks with the dominant leg. To be included in the study, each participant was required to have been training at the school for at least 12 months. The students were members of the school’s competitive soccer teams and, therefore, all participants had undergone standard systematic medical examinations on the basis of which they were deemed to be in good health and without any condition that could significantly impact the test results. Each subject provided consent for participation, and the research was approved by the management of FC Sloboda Tuzla (No. 01/1-sl./11).

### Variables

Fifteen variables were chosen for the evaluation of kinematic parameters. The following were considered independent and/or predictive variables: Sleg (length of the swing, from the starting phase to contact with the ball (cm)); Aknee1 (the angle of the knee joint in the starting phase of the swing of the swing foot (degrees)); Fspeed1 (the speed of the swing foot in the starting phase of the swing (m/s)); Fspeed2 (the speed of the swing foot in the middle of the swing (m/s)); Fspeed3 (the speed of the swing foot at the time of contact with the ball (m/s)); Kspeed1 (the speed of the knee of the swing foot in the starting phase of the swing (m/s)); Kspeed2 (the speed of the knee of the swing foot in the middle of the swing (m/s)); Kspeed3 (the speed of the knee of the swing foot at the time of contact with the ball (m/s)); HCG1 (the height of the body focus in the swing phase (cm)); HCG2 (the height of the body focus at the time of contact with the ball (cm)); Llast (the length of the last step (cm)); AsCG (the angle between the surface of the ball and the body focus (degrees)); SD (the distance from the support leg to the ball (foot posterior displacement; cm)); and Akbase (the knee angle of the support leg at the time of contact with the ball (degrees)). Additionally, we considered one dependent variable: Vball (the speed of the ball (m/s)).

### Procedures

All testing was conducted at the Exercise Science Laboratory of the Faculty of Physical Education and Sport, Tuzla University, from 10 am to 12 noon. Tests were completed on a parquet surface and the participants wore outfits that complied with the conditions of testing. The Ethical Committee of Tuzla University approved the study and the procedures conformed to the principles of human experimentation outlined in the Declaration of Helsinki.

The participants performed a 15min warm-up, which consisted of three phases. The first phase was a 3 min run (jogging, jumping, and dynamic stretching); the second phase consisted of exercises with a ball in pairs. During this phase participants passed the ball at different distances with various parts of the foot. All exercises were performed in motion; the final stage was for three participants in the formation of a triangle to change their position and shoot at the goal (crisscross). Testing commenced at the end of the warm-up.

The participants began the test by kicking with the dominant leg. Each kick was completed three times. The subjects rested for one minute between kicks, in order to prepare for the next kick. Each kick ended with a three-step run that started within 4 m from the ball and was at an angle less than 45° to the goal. The kick was performed at a distance of 10 m from the goal. Each kick was recorded by two synchronized cameras (Casio Ex-F1) that were positioned 12 m from the place of the kick, with a 90° angle between them (Picture 1).

The testing space was calibrated within a frame of 1 m x 1 m x 2 m (Picture 2). The cameras had a frequency of 300 Hz and a resolution of 720 x 576 pixels. Data processing was completed with the use of the APAS motion analysis system (Ariel Dynamics Inc., San Diego, CA).

### Statistical analysis

Multiple correlation analysis evaluates prediction of one criteria variable based on a defined group of prediction variables. Multivariate regression analysis provides the multiple correlation (R) and β coefficients, which are fundamental standardized coefficients of partial regression. The multiple correlation coefficient expresses the strength and interconnection of a group of predictors and criteria variables; it rates the value of the total system of prediction variables in the prediction of one criteria variable. Each of the variables within the prediction system has its own β coefficient. The higher this coefficient, the more impact the variable has on the prediction of the criteria variable. The β coefficient is similar to the partial correlation coefficient because it shows separate contributions of individual variables in defining common variations of the group of predictors and the criteria variable. In order to define the impact of the predictive group of variables on the criteria variable, we used the forward selection method of multiple regression analysis. This algorithm is used to create a variable (a predictor) through the linear combination of a series of predictor variables in order to yield the best possible approximation of the criteria variable. The first variable entered into the equation in the first step of the forward method of regression analysis is the predictor variable with the highest correlation with the criteria variable. This is followed by the introduction of the predictor variable with the highest partial correlation, which indicates the highest correlation with the criteria, when the parts they share with the first predictor variable are removed from the criteria and the predictor variable. The steps continue in this manner until all predictor variables with statistically significant partial correlation with the criteria have been used. Therefore, each next step controls the partial correlation for all the variables that have already been placed in the equation. In our study, three applied predictive variables that evaluated angle values were examined with the Bloom procedure. The Bloom procedure was used to transfer these variables to a higher level (proportional scale) in order to make their units of measurement consistent with the other variables.

## Results

Within the applied regression analysis, the predictor group of variables included the evaluation of kinematic parameters. The criteria variable was presented as the speed of the ball for each attempt and it was expressed in m/s. The results of the regression analysis are presented in Table 1. On the basis of our results, the regression analysis was terminated in the second step, where R = 0.690. The coefficient of multiple determination (R^2^), which represents the percentage of variability and was a joint criteria to the group of prediction variables in the second step, equalled 0.476.

Results of the F test within each regression analysis were used to test the validity of the regression models in each step. The models were statistically significant in each step (p = 0.01), which indicated that it was not possible to predict the criteria based on the models.

The values of the β coefficients were calculated for each of the steps of the regression analysis. Analysis of the individual impacts of predictor variables to the criteria revealed that two variables offered a significant portion of the prediction of the characteristic of the criteria variable. Fspeed3 was the first variable of the applied regression analysis that significantly predicted the criteria (p = 0.01). SD was the second variable, along with the partial correlation with the criteria variable, that had a statistically significant impact on the prediction of the criteria variable.

Table 2 presents the mean values of the variables Fspeed3, SD, and Vball, as well as the coefficients of correlation between these predictor variables and the criteria variable (the speed of the ball).

## Discussion

**Figure 1:**
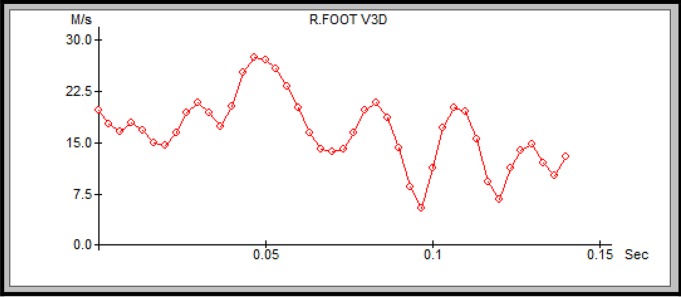
The speed of the swing foot (Fspeed3) at the time of contact with the ball

Impulse is the product of force and time, and it is logical to conclude that a higher speed of the foot at the moment of contact with the ball will produce a higher speed of the ball (Figure 1).

**Figure 2:**
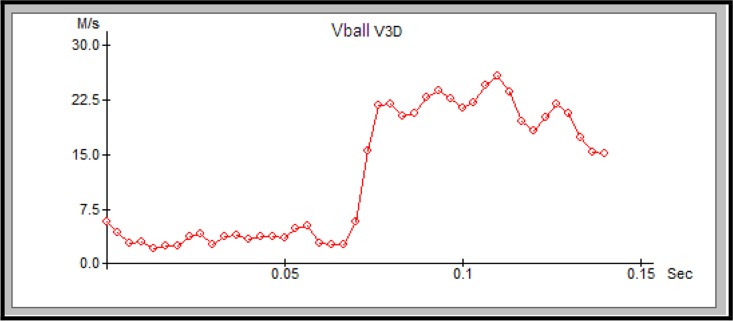
The speed of the ball (Vball) over time during an instep kick

The first of Newton’s Laws establishes the need for an outside force, which, in our study, was the leg kicking the ball, in order to change the status of the ball from a stationary object to a moving body. The achieved speed of the ball (Figure 2) depends on several factors: characteristics of the force (size, direction, and point of application) and the mass of the ball (Opavsky, 2000).

Our results clearly demonstrate that the velocity of the ball primarily depends on the speed of the kicking leg at the moment of contact with the ball and the position of the support leg when performing the kick (Tanaka et al, 2006). These two variables are interrelated. Specifically, a higher speed of the foot and a smaller distance between the ball and the support leg contribute to a higher speed of the ball. This is due to the creation of a high force impulse and, at the same time, an increase in the phases of compression and restitution (Opavsky, 2000; Droge et al., 2002; Asai et al., 2005). Dorge et al. (2002) reported that a high speed of the foot (18.6 m/s) and a high angular velocity (28.1 rad/s) led to a high coefficient of restitution (COR = 0.50), where a larger COR means a higher coefficient of elasticity of the ball and thus, a higher ball speed. Additionally, Asai et al. (2005) explained that a high force impulse (Ns = 11.06 ± 0.33 kg m/s) was associated with a high speed of the foot (23.12 ± 1.03 m/s) and a high speed of the ball (25.44 ± 0.76 m/s). Hussain and Arshad Bari (2012) defined a positive correlation between the speed of the foot and the speed of the ball (r = 0.94). Further, Poulmedis et al. (1998) stated that a high angular speed was positively correlated with the speed of the ball (r = 0.64 – 0.82).

Our results also show that positioning of the support foot has a significant impact on the achieved speed of the ball. Coefficients reported in Tables 1 and 2 (β coefficient = −0.269; r = −0.461) show that a large distance between the support foot and the center of the ball results in a lower speed of the ball (similarly, a small distance between the support foot and the center of the ball results in a high speed of the ball). The reason for the smaller speed when the support leg is positioned farther from the center of the ball is probably due to the fact that the impulse of the kicking force is directed to the center of the ball (Opavsky, 2000; Shinkai et al., 2009). Andersen et al. (2008) reported that the coefficient of restitution was highest at the lowest acceleration of the foot (11.9 m/s), when the force impulse was closer to the center of the ball. Conversely, the highest acceleration (16.1 m/s) occurred when the force impulse was directed farther away from the center of the ball.

Technically advanced players firm the kicking leg to the body at the moment of kicking the ball and, therefore, increase the amount of body mass that participates in the kick (Opavsky, 2000; Reilly, 2003). Despite other factors and variables, we acknowledge that the technical quality of the player is important in achieving an efficient kick. Shan et al. (2012) defined the connection of kinematic parameters with the speed of the ball based on biomechanical modeling of the entire body. The correlation coefficients for novice, advanced, and professional players were 0.754, 0.913, and 0.951, respectively. The correlation coefficients for novice, advanced, and professional female players were 0.728, 0.911 and 0.953, respectively. As previously stated, alterations in positioning of the support leg relative to the center of the ball contribute to different speeds of the ball. Positioning of the support leg is conditioned by the swing angle. Finally, different swing angles and different positions of the support leg result in different ball speeds. Some research showed that a swing angle of 45^°^ generates the maximum speed of the ball (Isokawa and Less, 1998). Scurr and Hall (2009) reported mean values of variations in positioning of the support leg and achieved speed of the ball at different swing angles, resulting in the highest average speed of the ball (supporting foot lateral displacement from the ball: 45^°^, 34.6 ± 6.1 cm, V ball 34.47 ± 2.12 m/s supporting foot posterior displacement from the ball: 45^°^, 11.3 ± 9.1 cm, V ball 34.47 ± 2.12 m/s).

Length of the final step in the swing determines acceleration or deceleration of the foot and can significantly impact the speed of the foot and, ultimately, the speed of the ball. Potthast et al. (2010) determined that substantial decelerations in the final step were correlated with a high speed of the ball (r = 0.60). Slowing of the center of the body contributes to the acceleration of the open end of the kinetic chain and, as such, leads to a high speed of the foot. The speed of the ball is also largely impacted by the position of the foot of the kicking leg. Ishii and Maruyama (2007) explained that the speed of the ball increased when the contact surface used to kick the ball was closer to the center of the foot of the kicking leg because the force (impact force 1200 N) resulted in an average highest speed of the ball (16.3 m/s). In the instep kick, the natural position of the body and foot allows for a high speed of the foot of the kicking leg and a high speed of the ball, unlike some other kicking techniques. Levanon and Dapnea (1998) determined that speed of the foot and speed of the ball are lower with the side-foot kick (foot speed 19.1 ± 1.1 m/s; ball speed 23.4 ± 1.7 m/s) than with the instep kick (foot speed 20.3 ± 1.0 m/s; ball speed 28.0 ± 2.1 m/s).

**Figure 3:**
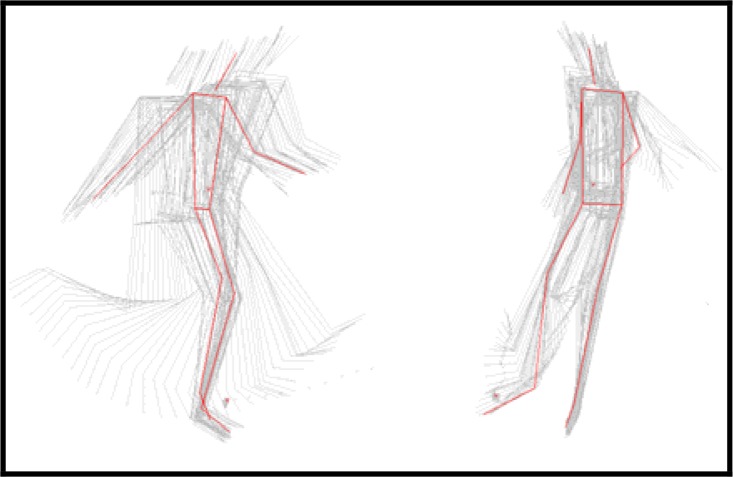
A kinogram of the kicking phase demonstrating the distance between the support foot and the bal.

On the basis of our results, we may conclude that there is an optimal position of the support foot that can lead to increased speed of the ball (Figure 3). Our findings confirm earlier research observations that a high speed of the ball is achieved if the support foot is positioned closer to the ball (Harrison and Mannering, 2006).

## Conclusion

This study suggests that there are two factors that contribute to increased speed of the ball in an instep kick in soccer, i.e. achieving a high speed of the foot, and positioning the support leg as close to the center of the ball as possible what allows the ball to be kicked with a large surface area of the foot that is close to the center of the foot.

Our conclusions are based on the correlations between observed kinematic parameters and the speed of the ball. Therefore, for a youth soccer player to successfully adopt this technique, he/she must pay attention to the technical elements presented in this paper.

Future research should be directed towards the application of electromyographic measurements and dynamometric measurements through a dynamometric platform. Additionally, future studies should include players in all soccer categories in a longitudinal study in order to correlate the findings with the technical qualities of the players and to strengthen the significance of the research.

## Figures and Tables

**Picture 1:**
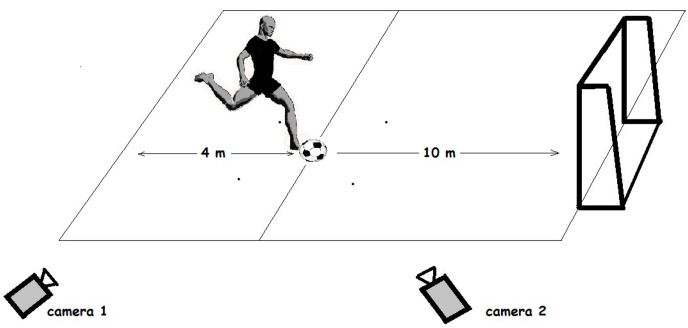
A scheme of the testing protocol

**Picture 2:**
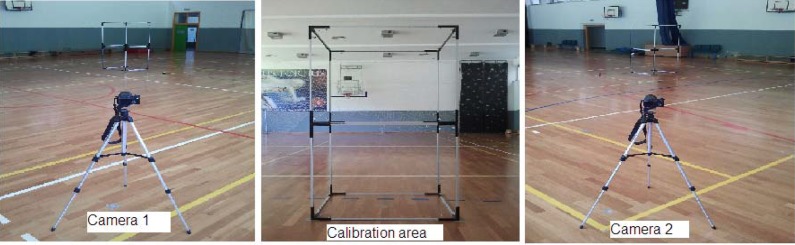
Space calibration

**Table 1:** Regression analysis (forward selection method)

Model		Coefficients (a)					Model Summary			ANOVA(c)	

		Unstandardize d Coefficients		Standardized Coefficients	T	Significance					

		B	Standard Error	β	B	Standard Error	R	R^2^	Standard Error	F	p

1	(Constant)	*8.637*	*2.440*		*3.540*	*.001*					
					
	Fspeed3 (the speed of the swing foot at the time of contact with the ball)	*.905*	*.178*	*.641*	*5.086*	*.000*	*.641(a)*	*. .411*	*1.911*	*25.867*	*.000 (a)*

2	(Constant)	*11.540*	*2.708*		*4.261*	*.000*					

	Fspeed3 (the speed of the swing foot at the time of contact with the ball)	*.784*	*.180*	*.556*	*4.365*	*.000*	*.690(b)*	*.476*	*1.827*	*16.371*	*.000 (b)*
					
	SD (the distance from the support leg to the ball (foot posterior displacement)	−*8.880*	*4.206*	−*.269*	−*2.111*	*.042*					

(a) predictors: (Constant), Fspeed3

(b) predictors: (Constant), Fspeed3, SD

(c) dependent variable: Vball (the speed of the ball)

**Table 2:** Descriptive statistics with the correlation coefficients

	Mean	Standard deviation	Vball
**Fspeed3** (the speed of the swing foot at the time of contact with the ball)	13.5997	1.74166	r =.641
**SD** (the distance from the support leg to the ball)	14.13	7.43	r =−.446
**Vball** (the speed of the ball)	20.9479	2.45784	
